# Nanopore long-read transcriptome sequencing reveals miR-22-3p-mediated transcriptomic remodeling and alternative splicing in chicken pectoral muscle

**DOI:** 10.1016/j.psj.2026.107769

**Published:** 2026-09-13

**Authors:** Jianing Yi, Jinyuan Chen, Jinjing Xu, Yingqiu Cai, Yi Zong, Mengmeng Run, Baoshun Qian, Qianhua Xie, Xiaolu Luo, Beibei Liu, Xingqing Zhang, Jiancheng Guo, Fei Ye, Hai Xiang, Hua Li, Zheng Ma

**Affiliations:** School of Animal Science and Technology, Foshan University, Foshan, Guangdong, 528225, China

**Keywords:** miR-22-3p, Nanopore long-read transcriptome, Alternative splicing, Chicken pectoral muscle

## Abstract

The full-length transcriptomic architecture mediated by gga-miR-22-3p in poultry muscle development remains largely unexplored. To address this, a lentiviral knockdown vector targeting miR-22-3p was constructed, and an in vivo miR-22-3p knockdown model was established in the pectoral muscle of 14-day-old Qingyuan partridge chickens through subalar injection. Oxford Nanopore Technologies (ONT) long-read RNA sequencing was employed to profile the full-length transcriptome and its remodeling under miR-22-3p knockdown. In total, 24,727 unannotated transcripts were identified. Differential expression analysis revealed 1,448 differentially expressed transcripts (DETs) and 535 differentially expressed genes (DEGs) that were significantly responsive to miR-22-3p knockdown. Gene set enrichment analysis (GSEA) showed that the downregulation of miR-22-3p was associated with significant suppression of muscle development-related pathways, including extracellular matrix (ECM)-receptor interaction, focal adhesion, and collagen metabolic processes, accompanied by the reduced expression of core genes such as *COL1A2, COL6A1*, and *EGF*. Moreover, alternative splicing analysis demonstrated that miR-22-3p knockdown induced extensive remodeling of splicing patterns and transcript isoform usage. This included splicing switches in key functional domains of *ENSGALG00000003955* and altered isoform ratios of *ESD* and *LOC101747587*, despite their stable gene-level expression. Taken together, these findings demonstrate that miR-22-3p may regulate the transcriptomic homeostasis of ECM and focal adhesion-related genes in chicken pectoral muscle by coordinating both gene expression levels and isoform-specific splicing. This study provides a full-length transcriptomic framework for understanding miR-22-3p-mediated post-transcriptional regulation during avian muscle development, offering new insights into the molecular regulation of poultry meat quality.

## Introduction

Poultry meat is a major source of high-quality animal protein worldwide, and consumer demand has gradually shifted from basic supply toward improved meat quality. With the widespread adoption of intensive production systems, elucidating the molecular mechanisms underlying meat quality traits is of great importance for the genetic improvement of elite local chicken breeds, such as Qingyuan partridge chickens (Henchion et al., 2021; Mo et al., 2023). Skeletal muscle growth and development are precisely regulated at multiple levels, including the proliferation and differentiation of myogenic cells, the composition of muscle fiber types, and the dynamic remodeling of the extracellular matrix (ECM) (Fry et al., 2017; Csapo et al., 2020). The ECM, mainly composed of collagen, laminin, and other structural components, provides physical support for muscle fibers and serves as a key biological determinant of meat tenderness, texture and water-holding capacity (Dubost et al., 2016; Wang et al., 2022). Accumulating evidence has demonstrated that diverse signaling molecules and non-coding RNAs play important roles in maintaining the homeostasis of the skeletal muscle microenvironment.

MicroRNAs (miRNAs) are a class of endogenous non-coding RNAs, generally 21–25 nucleotides in length, that regulate gene expression by promoting target mRNA degradation or inhibiting translation (O'Brien et al., 2018; Wang et al., 2018). Classical muscle-specific miRNAs, also known as MyomiRs, have been extensively studied in the regulation of myogenesis (Coenen-Stass et al., 2016; Gu et al., 2025). In recent years, the regulatory role of the highly conserved miR-22-3p in muscle homeostasis has attracted increasing attention (Li et al., 2021). In mammalian models, miR-22 has been shown to participate in cardiac hypertrophy, muscle atrophy, metabolic reprogramming, mitochondrial biogenesis, and autophagic flux (Diniz et al., 2017; De Oliveira Silva et al., 2020). Dysregulated miR-22 expression is also closely associated with enhanced muscle fibrosis (Tomasini et al., 2025). Although previous studies have suggested that miR-22-3p is involved in ECM remodeling and fibrotic progression, its global regulatory landscape in poultry muscle development and meat quality formation remains largely unclear (Huang et al., 2021).

Alternative splicing (AS) has been demonstrated to play crucial roles in vertebrate development, while its dysregulation is closely associated with complex diseases (Mazin et al., 2021; Marasco and Kornblihtt, 2023). Through AS, a single gene can generate multiple transcript isoforms with distinct structures and potentially divergent biological functions (Yang et al., 2016; Kjer-Hansen and Weatheritt, 2023). The broad application of second-generation short-read RNA sequencing has greatly accelerated the identification of splicing events. However, due to limited read length, most transcript structures inferred from short-read data rely heavily on computational reconstruction rather than direct observation, which restricts the accurate characterization of full-length transcripts and complex isoform architectures (Castaldi et al., 2022; Monzó et al., 2025). In contrast, Oxford Nanopore Technologies (ONT)-based long-read RNA sequencing enables the direct sequencing of full-length transcripts, providing a powerful approach for precise isoform annotation and alternative splicing analysis (Davidson et al., 2022; Zong et al., 2024). Nevertheless, long-read transcriptomic studies focusing on miRNA-mediated splicing regulation in poultry skeletal muscle remain scarce.

In the present study, Oxford Nanopore Technologies (ONT) long-read RNA sequencing was employed to systematically construct the full-length transcriptomic landscape of chicken pectoral muscle under miR-22-3p knockdown. Global transcriptome changes, isoform abundance dynamics, and alternative splicing remodeling induced by miR-22-3p inhibition were systematically characterized. The analysis revealed that a considerable proportion of transcriptomic variation was not solely attributable to changes in total gene expression but was also reflected in altered isoform usage, suggesting widespread transcript isoform switching. Further characterization of differentially expressed and alternatively spliced transcripts identified several hub genes and transcripts. In addition, numerous unannotated transcripts were identified and validated. These findings suggest that miR-22-3p may contribute to the transcriptional homeostasis of the skeletal muscle microenvironment by coordinating both gene-level expression and isoform-specific splicing regulation. This study expands our understanding of miRNA-mediated transcript regulation in avian skeletal muscle and provides candidate molecular targets for poultry breeding.

## Materials and methods

### Ethical statement

All animal experiments were reviewed and approved by the Experimental Animal Welfare and Animal Experiment Ethics Committee of Foshan University (approval no. FOSU202103-28). All procedures involving animals were performed in strict accordance with the guidelines of the Chinese Animal Protection Association.

### Construction and validation of the miR-22-3p lentiviral knockdown vector

To ensure biological safety and prevent replication-competent lentivirus or generation of replication-competent lentivirus, a four-plasmid lentiviral packaging system was used in this study. The self-inactivating lentiviral vector pcDNA3.1-exons4-CDS-mCherry, the packaging plasmids pMDLg/pRRE and pRSV-Rev, and the envelope plasmid pCMV-VSV-G were provided by Suzhou GenePharma Co., Ltd. The lentiviral knockdown vector targeting miR-22-3p and the corresponding negative control vector was co-transfected with packaging plasmids into HEK293T cells using transfection reagents according to the manufacturer’s protocol. Lentivirus-containing supernatants were collected, purified, and concentrated to obtain high-titer viral particles. The knockdown model was established by injection of the lentivirus into chickens, and the knockdown efficiency of miR-22-3p was evaluated by quantitative real-time PCR. Lentivirus preparation was performed by Suzhou GenePharma Co., Ltd. (Suzhou, China).

### Animals and tissue collection

A total of six 14-day-old female Qingyuan partridge chickens were randomly divided into two groups: the negative control group (NC) and the miR-22-3p knockdown group (I), with three birds in each group. Chickens in the NC group were administered 300 μL of LV3-NC lentivirus via subalar intravenous injection at a titer of 10^9^ TU/mL, whereas chickens in the knockdown group were administered 300 μL of miR-22-3p inhibitor lentivirus at the same titer. All birds were housed in cages under standard management conditions, with ad libitum access to feed and water. During the experiment, the indoor temperature was maintained at 30–32°C, relative humidity was controlled at 50–60%, and a 20-h light/4-h dark photoperiod was applied. Seven days after injection, birds were humanely sacrificed, and pectoral muscle tissues were rapidly collected. A portion of each tissue sample was fixed in 4% paraformaldehyde for subsequent histological analysis. The remaining tissues were transferred into 2-mL cryotubes, immediately frozen in liquid nitrogen, and then stored at −80°C until RNA extraction and sequencing.

### Total RNA extraction and qRT-PCR validation

Total RNA was extracted from pectoral muscle tissues using TRIzol reagent (Servicebio, Wuhan, China) according to the manufacturer’s instructions. RNA purity and concentration were assessed using a micro‑volume UV‑Vis spectrophotometer (Q5000, Quawell, USA). RNA samples with an A260/A280 ratio ≥ 1.8 were used for subsequent analyses. Reverse transcription was performed using the SweScript RT I First Strand cDNA Synthesis Kit (Servicebio, Wuhan, China). Quantitative real-time PCR was conducted to evaluate the knockdown efficiency of miR-22-3p, and relative expression levels were calculated using the 2^−ΔΔCt^ method. After successful establishment of the miR-22-3p knockdown model, qualified RNA samples were subjected to Oxford Nanopore Technologies long-read transcriptome sequencing by Beijing Biomarker Technologies Co., Ltd. (Beijing, China).

### Nanopore sequencing data processing and bioinformatics analysis

Raw RNA sequencing data were generated using the Nanopore sequencing platform. Raw reads were first filtered to remove reads shorter than 200 bp and reads with a quality score lower than 6. Full-length non-chimeric (FLNC) reads were identified based on the presence of the 5′ and 3′ primers. Clean reads were then aligned to the chicken reference genome (*Gallus gallus*, assembly GRCg6a) using minimap2 (v2.26). Following sequence deduplication, non-redundant transcript structures (GFF3 format) were compared against the reference genome annotation using gffcompare to identify unannotated novel genes and novel transcripts. Long non-coding RNAs (lncRNAs) and open reading frames (ORFs) were further predicted for the identified transcripts. Raw read counts for genes and transcripts were derived from the aligned BAM files using featureCounts (v2.0.8), and were subsequently normalized using DESeq2 (v1.38.3) for differential expression analysis. Differentially expressed genes (DEGs) and differentially expressed transcripts (DETs) between the NC and knockdown groups were identified using DESeq2 with the following thresholds: |log₂FC| > 1 and *P* < 0.05 (adjusted for multiple testing using the Benjamini-Hochberg procedure). Principal component analysis (PCA), Gene Ontology (GO) functional annotation, Kyoto Encyclopedia of Genes and Genomes (KEGG) pathway enrichment analysis, alternative splicing analysis, and gene set enrichment analysis (GSEA) were subsequently performed. GSEA was conducted using the clusterProfiler package (R) on the BMKCloud platform (https://www.biocloud.net, accessed on May 29, 2024). In addition, potential target genes of miR-22-3p associated with chicken breast muscle and meat quality traits were predicted using miRDB (https://mirdb.org, accessed on May 13, 2024).

### Identification of alternative splicing events and isoform switching events

The normalized transcript expression matrix was subjected to the Astalavista software (v3.2.1) for the identification of alternative splicing events (ASEs). The percent spliced-in (PSI) value was calculated for each ASE. ASEs with PSI values lower than 0.1 were excluded from downstream analysis. Differential alternative splicing events (DAS) were identified using the thresholds of |ΔPSI| > 0.1 and *P* < 0.05. Isoform switching events were analyzed using the IsoformSwitchAnalyzeR package (v2.8.0). The DEXSeq-based statistical method implemented in IsoformSwitchAnalyzeR was used to detect significant changes in isoform usage between the miR-22-3p knockdown and control groups. Isoform switching events were defined according to two criteria: an absolute differential isoform fraction (|dIF|) ≥ 0.1, indicating at least a 10% change in isoform usage, and an adjusted *P* < 0.05. The dIF value represented the difference in isoform usage between the treatment and control groups, whereas the statistical test incorporated biological variation within each group. Open reading frames (ORFs) of transcripts were predicted using the analyzeORF function, and the start and end positions of coding sequences were determined. Nucleotide sequences and corresponding protein sequences were extracted using the extractSequence function. To evaluate the potential functional consequences of isoform switching, protein domains were annotated using the Pfam database, and transmembrane helices were predicted using DeepTMHMM. These functional annotation results were then integrated into the IsoformSwitchAnalyzeR workflow to infer downstream biological consequences of transcript isoform switching.

### Protein-protein interaction network construction and hub gene screening

Differentially expressed genes were imported into the STRING database (version 12; http://string-db.org, accessed on October 20, 2025) to construct a protein-protein interaction (PPI) network. Protein interactions with a confidence score > 0.4 were retained for subsequent analysis. The PPI network was visualized using Cytoscape software (v3.9.1; https://cytoscape.org, accessed on October 20, 2025), and hub genes were screened using the cytoHubba plugin.

### RT-PCR validation of representative alternative splicing events

Five representative genes, including *IDH3A, IDH3B, LOC771456, LOC107049847*, and *EIF4EBP1*, were selected for RT-PCR amplification and gel electrophoresis validation. Specific primers were designed using NCBI Primer-BLAST (https://www.ncbi.nlm.nih.gov) and targeted common regions shared by different transcript isoforms (Supplementary Table A1). The expected product sizes were determined according to the predicted transcript structures. All primers were synthesized by Sangon Biotech (Shanghai, China). Reverse transcription and PCR amplification were performed using the HiFiScript gDNA Removal RT MasterMix Kit (CWBIO, Wuhan, China) according to the manufacturer’s instructions. PCR products were separated by agarose gel electrophoresis, and the band patterns were used to validate alternative splicing events of the selected genes.

### Statistical analysis

All experimental data were analyzed using IBM SPSS software version 20.0 (IBM Corp. Inc.2020, USA) and are presented as mean ± standard error of the mean (SEM). Data analysis and visualization were performed using R software (v4.3.3), Origin 2021, and GraphPad Prism (version 10.1.2).

## Results

### Establishment of the miR-22-3p knockdown model

To validate the efficiency of miR-22-3p knockdown in vivo, qRT-PCR was performed on pectoral muscle samples collected from lentivirus-treated chickens. MiR-22-3p expression was significantly reduced in the knockdown group (I) compared with the control group (NC) (*P* < 0.05), confirming the successful establishment of the miR-22-3p knockdown model for subsequent transcriptomic analyses (Fig. 1).

**Fig. 1:**
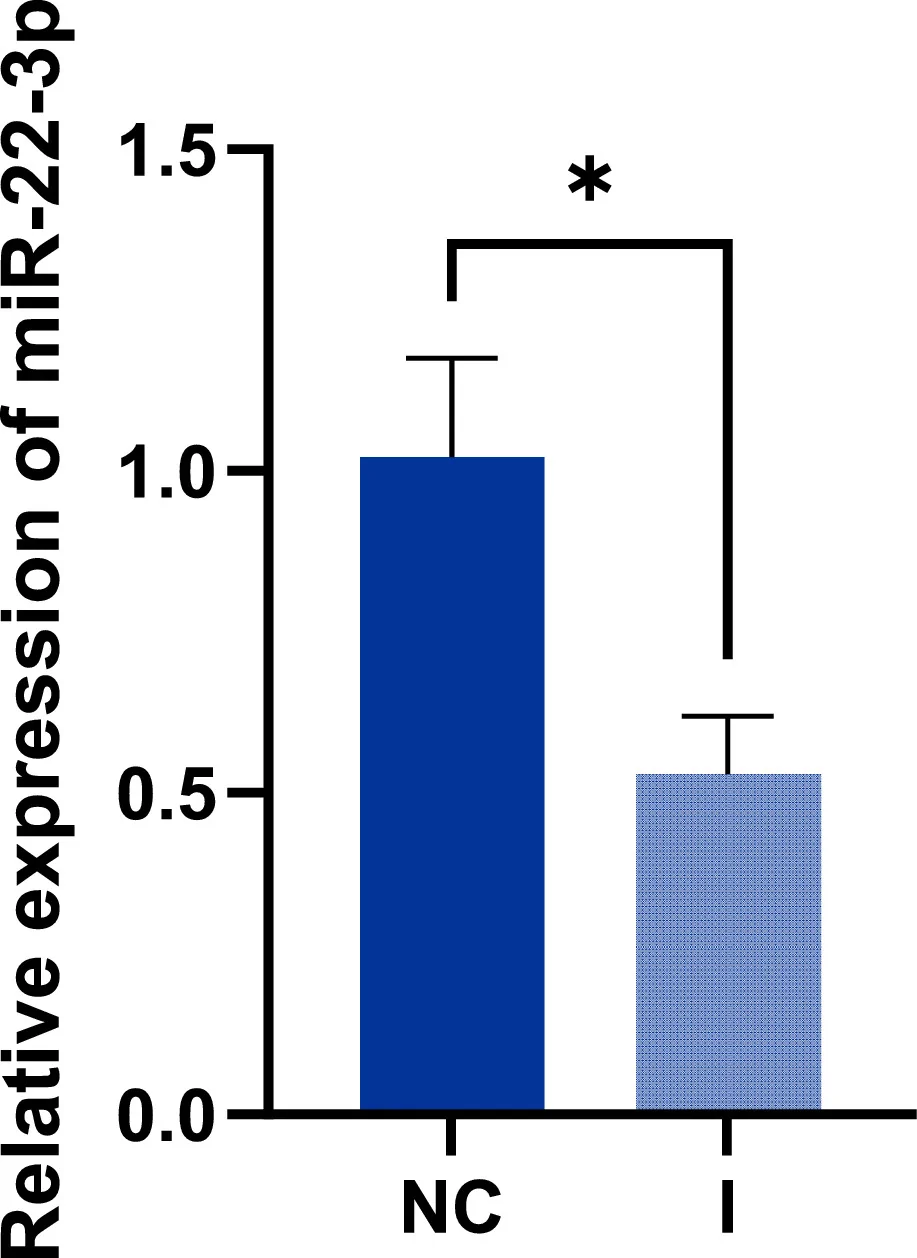
**Relative expression of miR-22-3p in the negative control (NC) and knockdown (I) groups (n** **=** **3), as determined by qRT–PCR.** Data are presented as mean ± SEM. Significant downregulation in group I is indicated (*P* < 0.05, two-tailed t-test).

### Landscape of the full-length transcriptome in chicken pectoral muscle

To characterize the full-length transcriptome landscape of chicken pectoral muscle, Oxford Nanopore Technologies (ONT) long-read RNA sequencing was performed on six pectoral muscle samples. Each sample generated at least 5.88 Gb of clean data (Table 1). In total, approximately 27.6 million long reads were obtained from all samples, with an average pass rate of 88.03% (Supplementary Table A2). The N50 values ranged from 1,449 to 1,621 nt across samples, and approximately 94.90% of long reads were mapped to the *Gallus gallus* reference genome (GRCg6a). After alignment and transcript collapsing, 34,757 non-redundant transcript sequences were identified (Supplementary Fig. B1). The global expression distribution of all samples was highly consistent, with similar median values and dispersion patterns, and no obvious batch effect was observed (Fig. 2A). These results indicated that the ONT sequencing data were of high quality and suitable for subsequent differential and splicing analyses.

**Table 1:** Summary of Oxford Nanopore sequencing data and mapping statistics.

Sample	Full-length reads	Clean Bases (bp)	N50 (nt)	Mean Length (nt)	Mapped rate (%)
I1	4,634,899	7,436,778,143	1484	1339	94.80%
I2	4,428,998	6,972,354,028	1482	1319	95.47%
I3	4,224,026	7,267,141,121	1621	1432	95.26%
NC1	4,120,664	5,876,956,598	1449	1206	94.30%
NC2	5,131,777	7,813,790,773	1466	1258	94.02%
NC3	5,113,961	7,874,790,773	1468	1293	95.53%

***Note:*** I = knockdown group; NC = negative control. Data mapped to the *Gallus gallus* reference genome (GRCg6a).

**Fig. 2:**
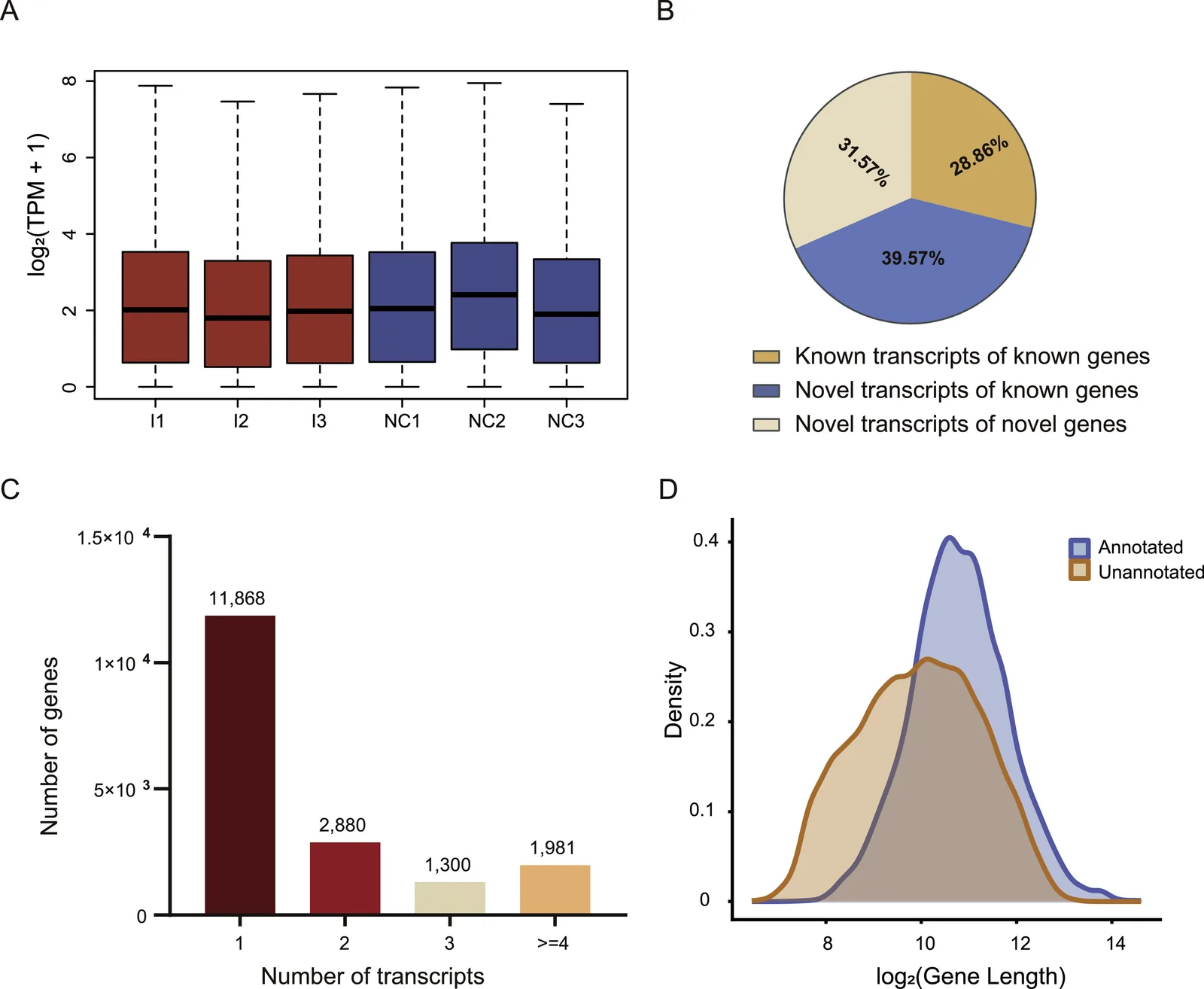
**Profiling of the chicken full-length transcriptome using Oxford Nanopore sequencing.** (A) Distribution of log₂(TPM + 1) across six samples, demonstrating high reproducibility. (B) Classification of 34,757 non-redundant transcripts into known transcripts of known genes (28.86%), novel transcripts of known genes (39.57%), and novel transcripts of novel genes (31.57%). (C) Distribution of isoform number per gene: 11% of genes (n = 1,981) express ≥4 isoforms. (D) Density plot of transcript lengths: annotated transcripts peak at ∼1,549 nt, while unannotated transcripts are significantly shorter (median = 1,217 nt vs. 1,382 nt, *P* < 2.2 × 10⁻¹⁶).

According to transcript annotation status, detected transcripts were classified into three categories: known transcripts of known genes (28.86%, n = 10,030), novel transcripts of known genes (39.57%, n = 13,753), and novel transcripts of novel genes (31.57%, n = 10,974) (Fig. 2B). Notably, 24,727 previously unannotated transcripts and 8,687 novel genes were identified. Transcript structure analysis showed that approximately 11% of detected genes (n = 1,981) expressed at least four transcript isoforms, suggesting widespread alternative splicing in chicken pectoral muscle (Fig. 2C). Compared with unannotated transcripts, annotated transcripts showed a relatively higher proportion of longer transcripts, with the highest density peak at approximately 1,549 nt (Fig. 2D). Furthermore, the median length of unannotated transcripts was significantly shorter than that of annotated transcripts (1,217 nt vs. 1,382 nt, *P* < 2.2 × 10⁻¹⁶). Collectively, these quality metrics demonstrated that the dataset provides a comprehensive full-length transcriptomic resource for chicken pectoral muscle and reveals a large number of previously unannotated transcripts.

### Differential expression of transcripts regulated by miR-22-3p

To take advantage of ONT long-read sequencing in capturing full-length transcripts at single-molecule resolution, differential expression analysis was performed at the transcript level. A total of 1,448 differentially expressed transcripts (DETs) were identified between the miR-22-3p knockdown group and the control group, including 971 upregulated and 477 downregulated transcripts (Fig. 3A; Supplementary Data 1). Hierarchical clustering of the top 20 DETs showed that biological replicates within each group grouped closely, whereas the knockdown group and the control group were clearly separated, with no obvious outlier samples (Fig. 3B). KEGG enrichment analysis of DETs revealed that miR-22-3p inhibition was associated with significant enrichment of pathways including glycerophospholipid metabolism, sphingolipid metabolism, ceramide/sphingomyelin synthesis, p53 signaling pathway, FoxO signaling pathway, and autophagy – animal (Fig. 3C; Supplementary Data 2). GO functional annotation further showed that DETs were mainly involved in biological processes and molecular functions such as cellular response to leucine starvation, neutral amino acid transmembrane transporter activity, and glycine, serine, and threonine metabolism. These enrichment results suggest that miR-22-3p knockdown mainly affects transcriptomic programs related to lipid metabolic remodeling and amino acid sensing or transport. Notably, these pathways are closely associated with muscle cell membrane homeostasis and the nutritional metabolic microenvironment. The altered abundance of these transcripts may result from changes in total gene expression or remodeling of intra-genic splicing patterns, prompting further analysis of alternative splicing regulation.

**Fig. 3:**
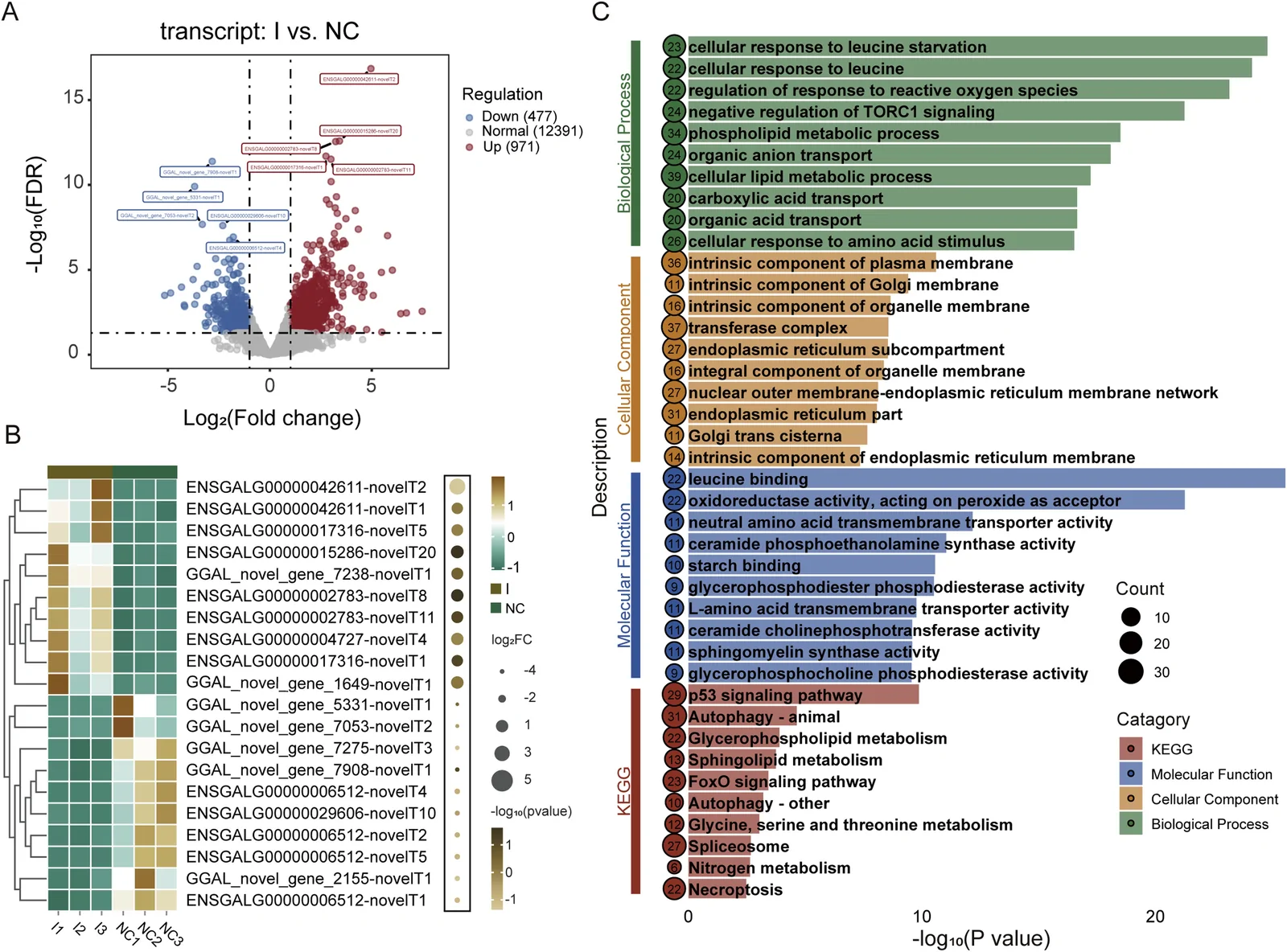
**Quantitative analysis of differentially expressed transcripts (DETs).** (A) Volcano plot of DETs (|log₂FC| > 1, FDR < 0.05); red and blue dots indicate upregulated and downregulated transcripts, respectively. (B) Hierarchical clustering heatmap of the top 20 DETs across all samples. Transcript IDs prefixed with GGAL_novel_gene_ represent novel genes identified by our ONT analysis pipeline that are absent from the Ensembl chicken reference annotation (*Gallus gallus*), whereas IDs starting with ENSGALG correspond to known gene loci in the Ensembl reference annotation. (C) Functional enrichment of DETs: significantly enriched GO terms and KEGG pathways, including autophagy, mTOR signaling, and response to leucine stimulus.

### MiR-22-3p regulates alternative splicing and isoform usage patterns

Based on the full-length transcriptome data, miR-22-3p-regulated differential alternative splicing events were systematically identified. Seven types of alternative splicing events were detected, including alternative 3′ splice site (A3SS), alternative 5′ splice site (A5SS), alternative first exon (AFE), alternative last exon (ALE), mutually exclusive exon (MXE), retained intron (RI), and skipped exon (SE) (Fig. 4A; Supplementary Data 3). The proportions of different splicing types were generally comparable between the knockdown and control groups. Among them, AFE was the most frequent splicing type across samples, whereas ALE, MXE, and RI occurred at relatively lower frequencies. In total, 8,901 and 509 alternative splicing events (ASEs) were detected in annotated genes and unannotated genes, respectively (Fig. 4C). Differential alternative splicing events were further screened using the criteria of |ΔPSI| > 0.1 and *P* < 0.05, resulting in 139 differential alternative splicing (DAS) events. Single-sample analysis indicated that AFE events were predominant. Combined analysis further showed that AFE events accounted for the highest proportion of splicing events in annotated genes (33.5%), whereas AFE accounted for 21.4% of events in unannotated genes. By contrast, ALE events were more frequent in unannotated genes (15.5%) than in annotated genes (4.8%). This marked difference in splicing composition suggests that unannotated transcripts may be generated through distinct splicing patterns and regulatory mechanisms.

**Fig. 4:**
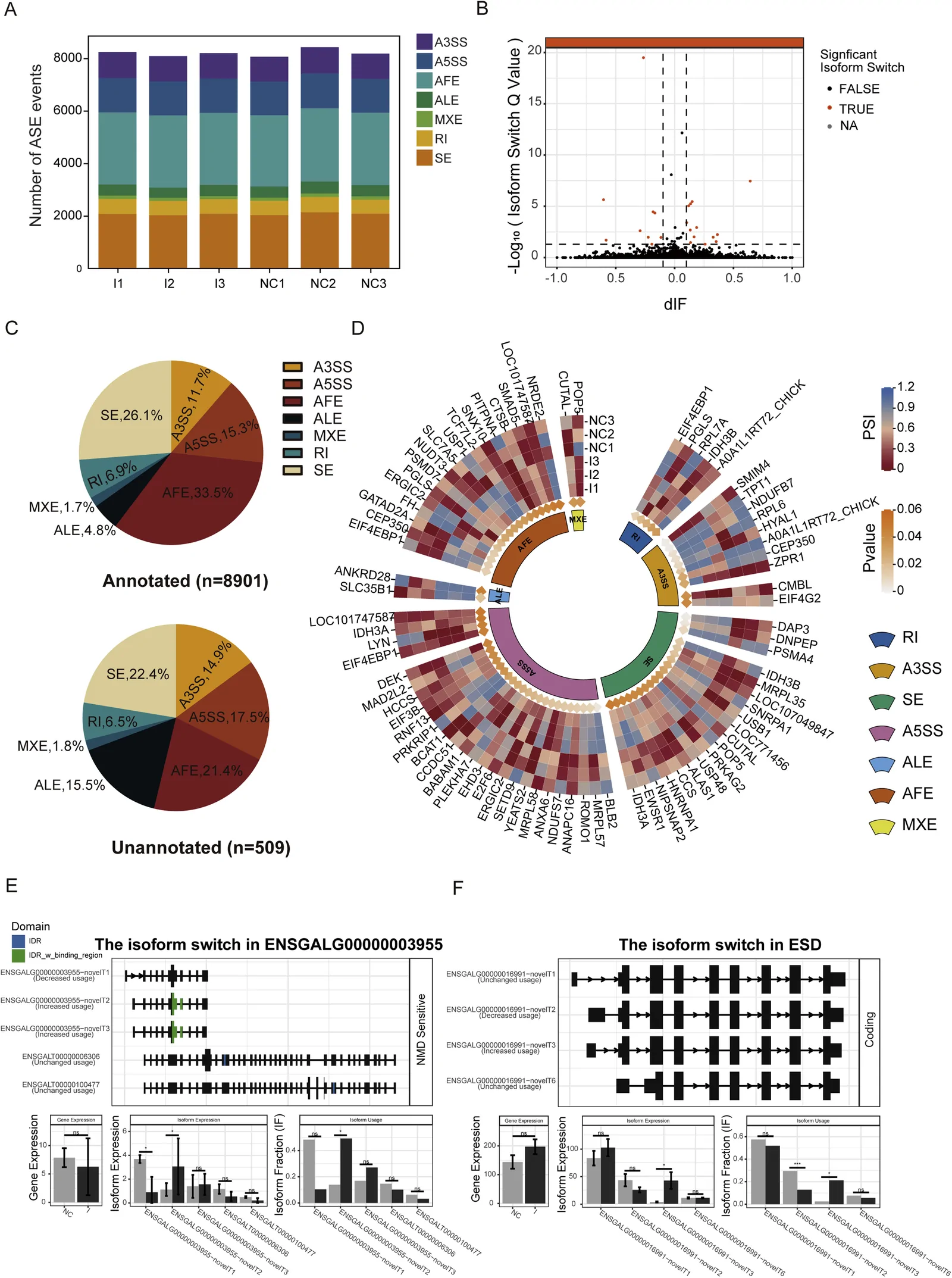
**Alternative splicing (AS) landscape and isoform switching under miR-22-3p knockdown.** (A) Distribution of seven AS types across samples, with Alternative First exon (AFE) being the most frequent. (B) Volcano plot of transcript usage change (dIF): significant isoform switches (|dIF| ≥ 0.1, *P* < 0.05) are highlighted. (C) Comparison of AS type proportions between annotated and unannotated genes. (D) Circular heatmap showing percent spliced-in (PSI) values of representative genes that exhibit differential alternative splicing events associated with muscle quality. (E, F) Representative isoform switching cases for *ENSGALG00000003955* and *ESD*: track plots show transcript structures and domains (e.g., IDR-binding regions); bar graphs show total gene expression vs. individual isoform fraction (IF) .

To investigate changes in isoform usage within the same gene, significant isoform switching events were detected by comparing isoform fraction values between the knockdown and control groups. Events with |dIF| ≥ 0.1 and adjusted *P* < 0.05 were considered significant. A total of 20 significant isoform switching events, involving 22 differentially expressed transcripts from 17 genes, were identified (Fig. 4B; Supplementary Data 4). Representative genes with differential alternative splicing events included *EIF4EBP1*, which is involved in translational initiation regulation; *IDH3B*, a key enzyme in energy metabolism; and *LOC101747587*. These genes showed significant shifts in exon inclusion levels after miR-22-3p inhibition (Fig. 4D). The presence of AFE, SE, and RI events in core functional genes further suggests that miR-22-3p not only regulates overall gene expression but also fine-tunes isoform composition at the post-transcriptional level, thereby potentially reshaping pectoral muscle development and metabolic homeostasis.

Several genes showed significant isoform switching without corresponding changes at the total gene expression level. For example, *ENSGALG00000003955* exhibited no significant difference in overall gene expression between the two groups, but its isoform usage pattern changed markedly. Specifically, the usage proportion of the novelT2 transcript was significantly increased in the knockdown group (*P* < 0.05), whereas the major transcript novelT1 was significantly downregulated at the expression level but showed no statistically significant change in usage proportion (*P* > 0.05). Notably, this switch was accompanied by changes in functional domain composition, as the increased novelT2 and novelT3 transcripts contained specific intrinsically disordered region (IDR)-binding regions, whereas novelT1 lacked these regions. In addition, structural annotation classified novelT1, novelT2, and novelT3 as NMD (nonsense-mediated decay)-sensitive transcripts (Fig. 4E). These results suggest that miR-22-3p knockdown alters both the domain structure of the encoded protein and the expression of NMD-sensitive isoforms. Similarly, the *ESD* gene (*ENSGALG00000016991*) showed typical isoform proportion switching in the knockdown group despite stable total gene expression. The usage proportion of novelT2 was significantly decreased, whereas both the usage proportion and absolute expression level of novelT3 were significantly increased (Fig. 4F). These results indicate that transcript isoform reorganization can mediate precise functional regulation independent of gene-level expression changes.

### Expression and ORF features of unannotated transcripts

To systematically characterize the molecular features of unannotated transcripts, expression levels and open reading frame (ORF) length distributions were compared between annotated and unannotated transcripts. Unannotated transcripts showed significantly higher expression levels than annotated transcripts (Fig. 5A), indicating that these newly identified transcripts were not merely low-abundance transcriptional noise. Further ORF analysis revealed clear differences between annotated and unannotated transcripts (Fig. 5B). ORFs of annotated transcripts were mainly distributed in the 250–750 nt and 750–1,250 nt intervals, consistent with the features of known protein-coding transcripts. In contrast, ORFs of unannotated transcripts were generally shorter and mainly concentrated in the 0–250 nt interval, accounting for approximately 25%, with the proportion gradually decreasing as ORF length increased. These results suggest that many unannotated transcripts may lack long ORFs and potentially function through non-coding regulatory mechanisms. To further explore the potential biological roles of unannotated transcripts, KEGG enrichment analysis was performed. The results showed that unannotated transcripts were significantly enriched in multiple pathways closely related to muscle biology, including mitophagy, oxidative phosphorylation, autophagy, ubiquitin-mediated proteolysis, and the mTOR signaling pathway (Fig. 5C). These pathways are involved in energy metabolism, protein homeostasis, and cytoskeletal maintenance in muscle cells. Notably, the enriched pathways of unannotated transcripts were highly consistent with those of annotated transcripts, further supporting the hypothesis that unannotated transcripts may actively participate in core regulatory processes associated with muscle development and meat quality (Supplementary Fig. B2). Collectively, these newly identified unannotated transcripts are characterized by distinct ORF features and significant enrichment in muscle-related functional pathways, suggesting potential roles in meat quality regulation through non-coding mechanisms.

**Fig. 5:**
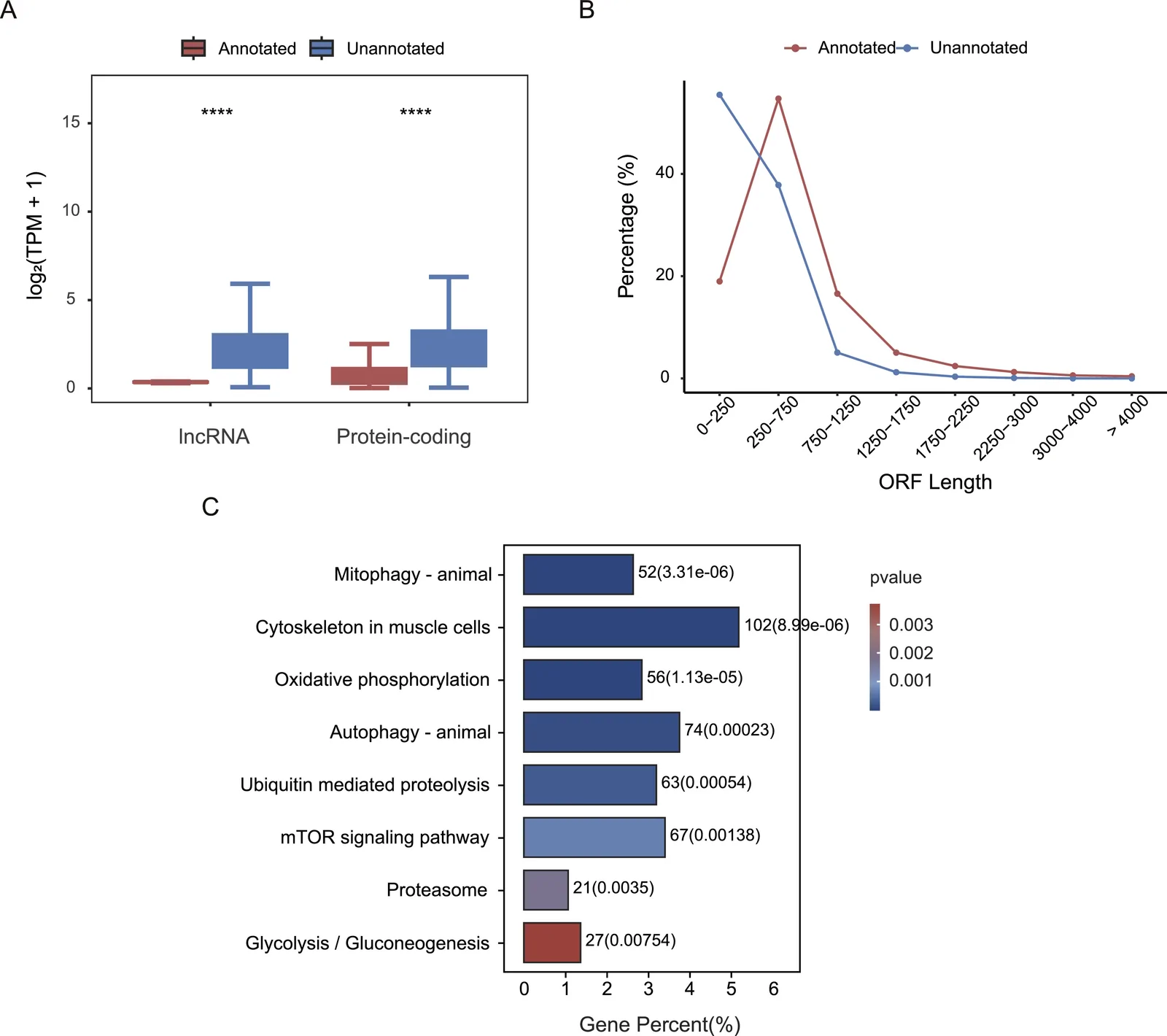
**Structural and functional characterization of unannotated transcripts.** (A) Expression comparison (log₂TPM + 1) of annotated and unannotated transcripts; unannotated transcripts show significantly higher median expression than annotated ones (*P* < 0.0001). (B) ORF length distribution: unannotated transcripts exhibit a shift toward shorter ORFs (<250 nt). (C) KEGG enrichment of unannotated transcripts: top pathways include mitophagy, oxidative phosphorylation, and ubiquitin-mediated proteolysis.

### Gene-level dysregulation, PPI network, and miR-22-3p target prediction

To further reveal how transcript-level and splicing alterations affect biological processes in pectoral muscle development, gene-level expression changes were integrated for downstream analysis. Using the thresholds of adjusted *P* ≤ 0.05 and∣log_2_FC∣≥1, a total of 535 differentially expressed genes (DEGs) were identified, including 372 upregulated and 163 downregulated genes (Fig. 6A; Supplementary Data 5). Gene set enrichment analysis based on KEGG and GO databases showed that miR-22-3p inhibition was negatively enriched in multiple pathways closely related to skeletal muscle structure and the cellular microenvironment. At the KEGG pathway level, focal adhesion (map04510, NES = −1.79, FDR < 0.05; Fig. 6C) and ECM-receptor interaction (map04512, NES = −2.03, FDR < 0.05; Fig. 6D) were significantly downregulated. GO enrichment analysis further showed significant negative enrichment of extracellular matrix structural constituent (GO:0005201, NES = −2.42, FDR < 0.01; Fig. 6E) and collagen-containing extracellular matrix (GO:0062023, NES = −2.75, FDR < 0.01; Fig. 6F). Leading-edge gene analysis revealed that collagen family genes, including *COL1A1, COL1A2, COL6A1, COL6A2, COL6A3*, and *COL4A2*, together with *ITGB1, FN1*, and *EGF*, were the major contributors driving the downregulation of these pathways. These findings suggest that miR-22-3p plays an important role in maintaining ECM homeostasis and cell-matrix interactions in pectoral muscle, and its inhibition may remodel the muscle microenvironment, affecting meat tenderness and water-holding capacity.

**Fig. 6:**
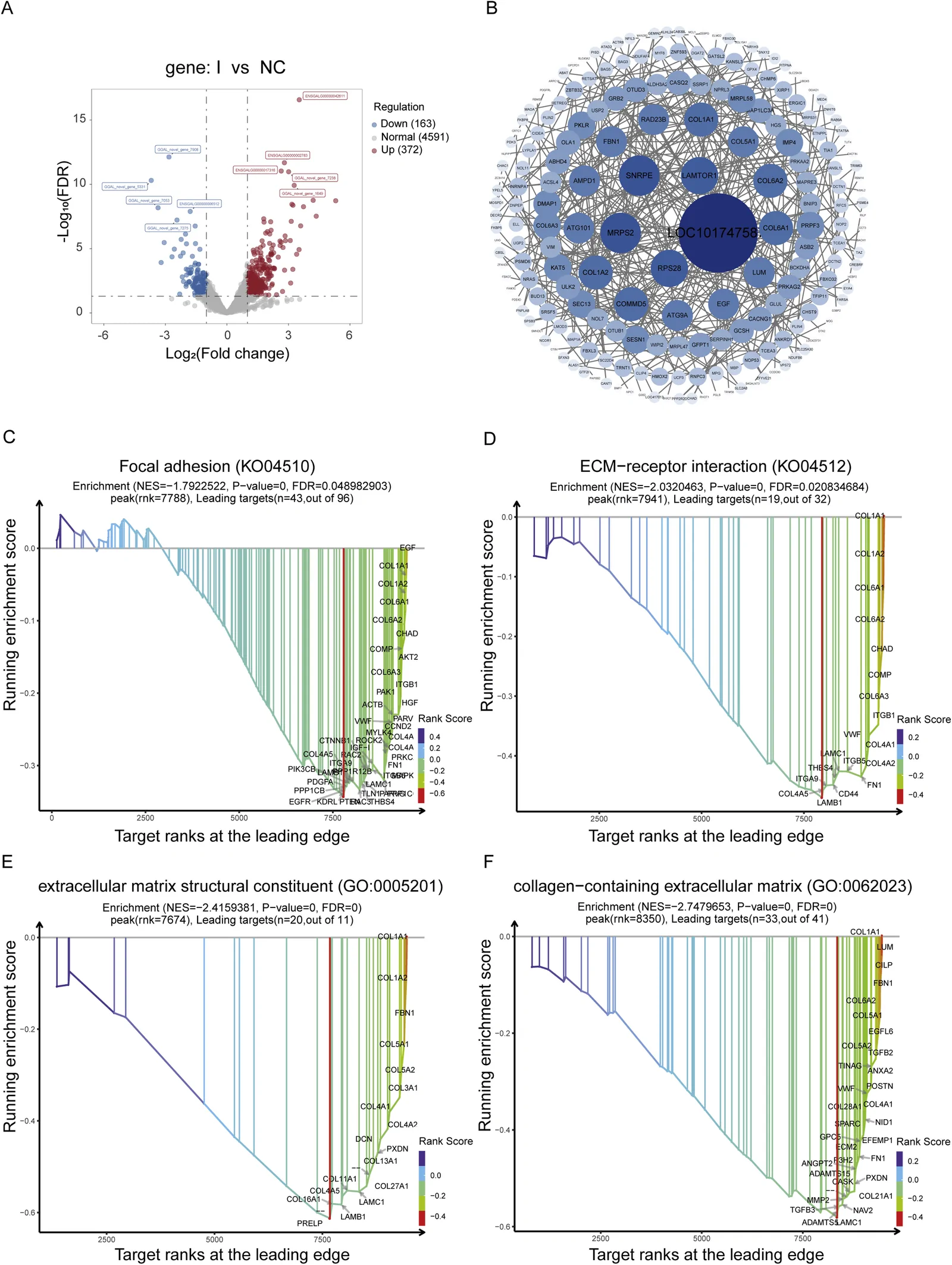
**Hub gene identification and pathway activation via PPI network and GSEA.** (A) Gene-level volcano plot (∣log_2_FC∣≥1, *P* < 0.05). (B) Protein-protein interaction (PPI) network of all differentially expressed genes (DEGs); the central node *LOC101747587* serves as a primary hub. (C–F) GSEA plots illustrating the significant suppression (negative enrichment) of pathways and gene sets upon miR-22-3p knockdown: (C) Focal adhesion (NES = −1.79, FDR = 0.049); (D) ECM-receptor interaction (NES = −2.03, FDR = 0.021); (E) extracellular matrix structural constituent (NES = −2.42, FDR < 0.001); and (F) collagen-containing extracellular matrix (NES = −2.75, FDR < 0.001). Negative NES values indicate that these structural and environmental pathways are significantly downregulated in the knockdown group.

To further explore interactions among DEGs and identify key genes involved in miR-22-3p-mediated regulation, a protein-protein interaction (PPI) network was constructed (Fig. 6B). The PPI analysis indicated that *LOC101747587, SNRPE, MRPS2, LAMTOR1, RAD23B, LUM, EGF, RPS28, FBN1, COL1A1, COL1A2, COL6A2, ATG9A, ATG101, AMPD1*, and *COMMD5* were associated with the regulatory effects of miR-22-3p. Based on centrality algorithms, 11 hub genes were identified, including *COL1A2, LOC101747587, MRPS2, LAMTOR1, SNRPE, RPS28, ATG9A, EGF*, SEC*13, SESN1*, and *IMP4*. Intersection analysis between these hub genes and predicted miR-22-3p target genes identified *EGF* as a potential direct target (Supplementary Fig. B3; Supplementary Data 6). Notably, *LOC101747587* was the only hub gene simultaneously regulated by differential expression and differential alternative splicing, accompanied by significant upregulation of multiple unannotated transcripts, including novelT1, novelT6, and novelT11. This suggests that *LOC101747587* may occupy a central position in miR-22-3p-mediated regulation of chicken pectoral muscle development. By contrast, the remaining hub genes, including *COL1A2* and *EGF*, mainly showed changes in total gene abundance without significant isoform switching.

### RT-PCR validation of representative alternative splicing events

To validate the differential alternative splicing events identified by ONT full-length transcriptome sequencing, five representative genes, including *IDH3A, IDH3B, LOC771456, LOC107049847*, and *EIF4EBP1*, were selected for RT-PCR amplification and gel electrophoresis analysis (Fig. 7). The results showed that two novel transcripts of *IDH3A* and *LOC771456* were detectable in the knockdown group, whereas their band intensities did not show obvious changes, consistent with the sequencing prediction that these transcripts were not significantly altered. For *IDH3B*, two novel transcripts, *IDH3B-novelT1* (*ENSGALG00000037401-novelT1*) and *IDH3B-novelT5*, produced two clear bands in the knockdown group with sizes corresponding to the predicted transcript structures. The intensity of the novelT1 band decreased in the knockdown group, whereas the novelT5 band remained stable in both groups, suggesting an isoform switch. For *LOC107049847*, novelT1 and novelT2 showed similar expression changes, with both bands markedly enhanced in the knockdown group. For *EIF4EBP1*, among the three novel transcripts (novelT6, novelT8, and novelT9), only novelT8 was enhanced in the knockdown group, whereas the other bands remained relatively stable. These validation results support the reliability of the ONT-based alternative splicing analysis and indicate that miR-22-3p may modulate meat quality-related biological processes by precisely regulating isoform ratios of selected core genes in chicken pectoral muscle.

**Fig. 7:**
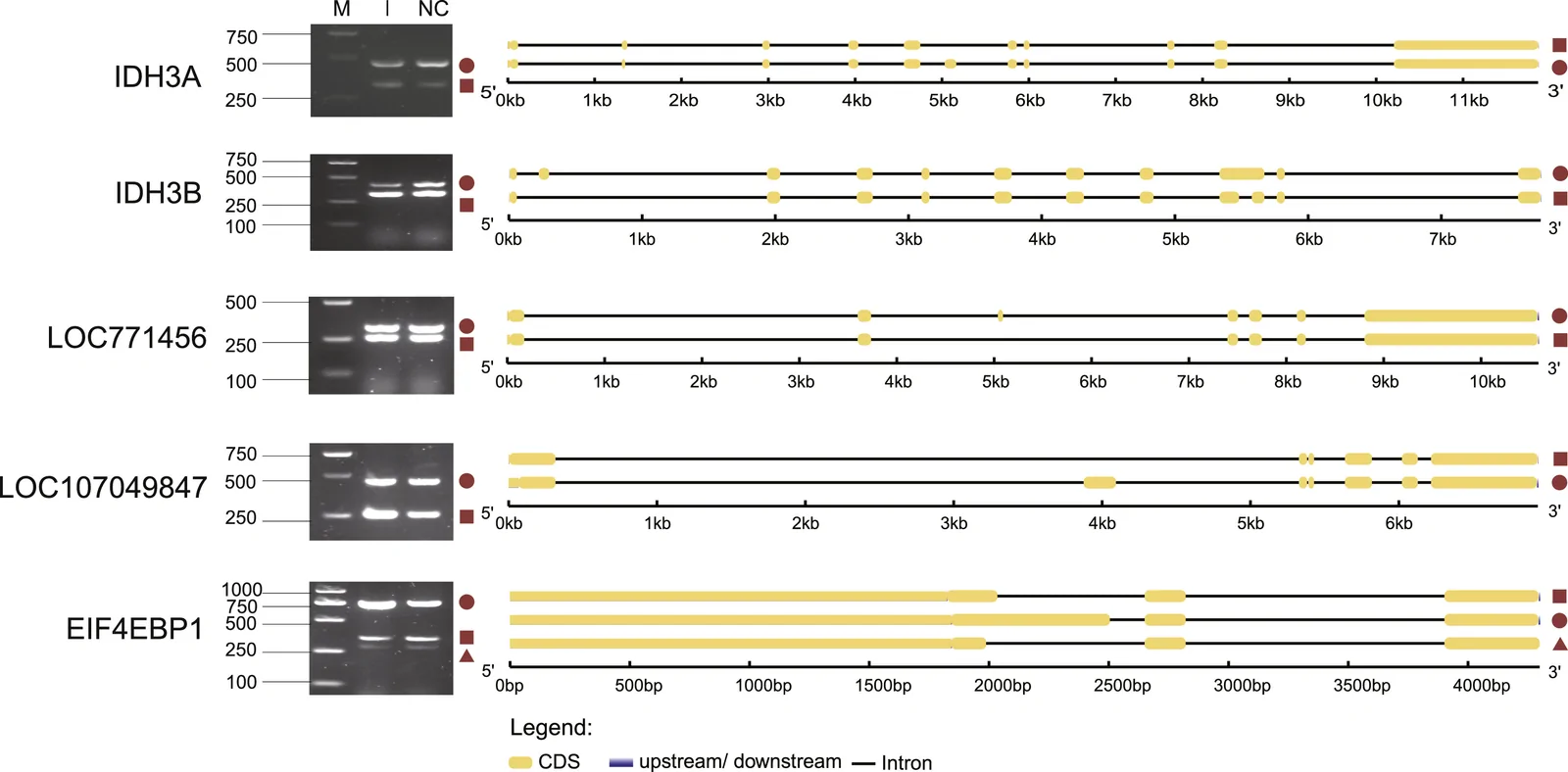
**RT-PCR validation of differentially spliced isoforms.** (A) Agarose gel electrophoresis confirming the presence of spliced isoforms for 5 representative genes (*IDH3A, IDH3B, LOC771456, LOC107049847*, and *EIF4EBP1*). Numbers beside markers indicate molecular weight (bp). (B) Schematic diagrams of genomic structures: exons (blue boxes) and introns (lines). Red symbols (circles/stars) indicate novel transcripts (novelT1–T9) identified by ONT sequencing. Corresponding intensity shifts between NC and I groups validate the isoform switching predicted by the transcriptome analysis.

## Discussion

In this study, Oxford Nanopore Technologies (ONT) long-read RNA sequencing was used to characterize the full-length transcriptome of chicken pectoral muscle following miR-22-3p inhibition, enabling the investigation of miR-22-3p-mediated transcriptomic regulation in poultry skeletal muscle. In the present analysis, a total of 24,727 unannotated transcripts were identified, a number comparable to previous long-read transcriptomic studies in livestock and poultry species (Guan et al., 2022; Shu et al., 2022; Jia et al., 2024; Wu et al., 2024; Cao et al., 2025). The large number of unannotated transcripts indicates that the complexity of the poultry muscle transcriptome has been substantially underestimated by short-read RNA sequencing. Traditional next-generation RNA sequencing largely depends on reference genome annotation and computational transcript assembly, which limits its ability to accurately reconstruct complex isoforms. In contrast, ONT long-read sequencing directly captures full-length RNA molecules, thereby improving the identification of transcript isoforms and revealing numerous transcripts that may have been overlooked in previous studies (Byrne et al., 2017; Kuo et al., 2020; Glinos et al., 2022; Sun et al., 2024). In addition, 1,448 differentially expressed transcripts were identified, suggesting that miR-22-3p inhibition induces extensive transcriptomic remodeling at the isoform level. These findings provide a global full-length transcriptomic perspective for a better understanding of the fine post-transcriptional regulation mediated by miRNAs in poultry muscle.

Skeletal muscle structural remodeling is a key biological basis underlying meat quality traits (Listrat et al., 2016; Picard and Gagaoua, 2020; Matarneh et al., 2021; Mo et al., 2023). During this process, growth factors and extracellular matrix components, including *EGF* and *COL1A2*, form regulatory networks that influence muscle microenvironment homeostasis (Csapo et al., 2020; Teng et al., 2025). In this study, GSEA showed that miR-22-3p inhibition led to significant negative enrichment of pathways related to collagen trimer assembly, myofibril structure maintenance, focal adhesion, and ECM-receptor interaction. These results are consistent with previous findings that miR-22 participates in fibrotic regulation in mammalian models (Tomasini et al., 2025). Among the identified hub genes, *COL1A2* showed the most significant downregulation, and multiple full-length isoforms of *COL1A2* exhibited decreased expression. *COL1A2*-mediated collagen deposition has been closely associated with the stiffness of the endomysium and perimysium, thereby affecting meat tenderness and other meat quality traits (Li et al., 2022; Roy and Bruce, 2024). Therefore, the downregulation of *COL1A2* may reduce excessive collagen deposition and muscle connective tissue stiffness, exerting favorable effects on meat tenderness. In addition, the upregulation of hub genes such as *RPS28* suggests that miR-22-3p may also influence muscle homeostasis by modulating protein synthesis and degradation fluxes (Jiao et al., 2021).

In addition to global transcriptional changes, stable alterations in splicing patterns further revealed the precision of miR-22-3p-mediated regulation. A total of 139 differential alternative splicing events were identified after miR-22-3p inhibition. For *ENSGALG00000003955*, miR-22-3p knockdown promoted a shift toward NMD-sensitive transcripts, pointing to a potential splicing-dependent gene silencing mechanism independent of total expression changes (Marasco and Kornblihtt, 2023). Meanwhile, *ESD* displayed isoform switching through altered relative isoform abundance despite stable total gene expression. In terms of splicing type distribution, alternative first exon events accounted for the largest proportion, a pattern that has also been reported in multiple vertebrates, including pigs and chickens (Tapial et al., 2017; Hu et al., 2021; Wang et al., 2024). These results suggest that miR-22-3p not only regulates muscle structural remodeling through global expression changes of hub genes, but also fine-tunes the functions of specific genes by modulating alternative splicing and isoform usage. Notably, full-length transcriptome analysis revealed a distinct regulatory pattern: core hub genes such as *COL1A2, EGF*, and *RPS28* mainly exhibited changes in total expression abundance without significant isoform switching. This finding is consistent with integrative transcriptomic and splicing analyses of skeletal muscle in pigs with different intramuscular fat contents, where only a small number of genes were simultaneously differentially expressed and differentially spliced, whereas most functional genes contributed to meat quality formation primarily through expression-level regulation rather than alternative splicing (Hao et al., 2022).

The identification of numerous unannotated transcripts by ONT long-read sequencing provides new insights into the regulatory architecture of the muscle metabolic microenvironment. These unannotated transcripts, including novel isoforms of the hub gene *LOC101747587*, were significantly enriched in pathways related to mitophagy, oxidative phosphorylation, and mTOR signaling. Their enrichment characteristics are consistent with regulatory mechanisms previously reported in skeletal muscle studies of livestock species such as yak (Muniz et al., 2022; Chen et al., 2024). In particular, *LOC101747587* showed significant changes across multiple unannotated isoforms, suggesting that previously uncharacterized isoforms of this gene may function as regulatory switches in muscle metabolism. Given the close association between mitochondrial autophagy, oxidative metabolism, and meat flavor formation, these transcripts may influence meat quality by modulating the intensity of oxidative metabolism in muscle cells (Jin et al., 2021). Intriguingly, unannotated transcripts exhibited a higher overall median expression level than annotated transcripts. This pattern may be partly attributed to dataset composition and bioinformatic filtering criteria. The annotated group encompasses a broad spectrum of genes with low basal expression across the genome that could lower its overall median. In contrast, low-coverage transcriptional noise in the unannotated category tends to be excluded during stringent bioinformatic quality control. Consequently, the detected unannotated transcripts likely derive from transcriptionally active genomic loci in skeletal muscle or comprise tissue-abundant regulatory RNAs with short ORFs, suggesting that they are not merely low-abundance transcriptional noise, but potentially represent functional regulatory elements in poultry skeletal muscle.

Although this study systematically revealed the impact of miR-22-3p inhibition on the full-length transcriptome of chicken pectoral muscle, several limitations should be acknowledged. Technically, ONT sequencing has a relatively high raw read error rate, which can be improved through optimized base-calling algorithms and correction with second-generation short-read sequencing data (Zhang et al., 2020; Holley et al., 2021; Wick et al., 2023). While short-read hybrid error correction was not performed in the present study, the high genome mapping rate (>94%) and deep sequencing coverage across biological replicates (Table 1) helped guarantee the confidence of unannotated transcript identification. The use of poly(A)-selected libraries may also result in relatively low coverage of some non-polyadenylated non-coding RNAs, limiting the comprehensive characterization of non-coding RNA-mediated regulation (Zhao et al., 2018). In terms of experimental validation, subalar intravenous delivery leads to systemic lentivirus spread, and our preliminary assessment showed that miR‑22‑3p levels were modulated in multiple organs including the heart and liver. As the current study focuses primarily on pectoral muscle transcriptomic responses, comprehensive characterization of biological consequences in these extra‑pectoral tissues has not yet been performed, which remains to be systematically investigated in future work. Additionally, due to the limited sample size and biological variation, several classical miR-22 target genes did not reach statistical significance in the present analysis, and the direct targeting relationships between miR-22-3p and potential novel targets, especially newly identified unannotated transcripts, remain to be experimentally validated. Future studies integrating larger-scale phenotypic measurements and molecular interaction experiments will help elucidate the biological functions of miR-22-3p-regulated transcripts and provide valuable candidate targets for molecular improvement of poultry meat-quality traits.

## Conclusion

In summary, this study constructed a full-length isoform map of Qingyuan partridge chicken pectoral muscle under miR-22-3p inhibition using ONT long-read transcriptome sequencing. The resulting transcriptome dataset provides a reference resource for investigating muscle development and meat quality traits at the isoform level in poultry. The integrated transcriptomic analysis indicates that miR-22-3p is closely associated with muscle tissue architecture and isoform regulation. These regulatory effects may further influence key cellular processes and structural features in skeletal muscle. Overall, this transcriptomic evidence suggests that miR‑22‑3p may contribute to muscle phenotypic variation via the coordinated regulation of gene expression and alternative splicing. These full-length transcriptomic data not only provide high-quality candidate targets for molecular-assisted breeding aimed at improving poultry meat quality, but also offer important evidence for understanding the post-transcriptional regulatory logic of miRNAs in vertebrates.

## Author contributions

**Jianing Yi:** Implemented the study, performed the Nanopore long-read sequencing pipeline and full-length transcriptome reconstruction. Elucidated the regulatory mechanism by which miR-22-3p modulates muscular alternative splicing and isoform switching. Drafted the full manuscript and handled all supplementary experiments and reviewer responses during revision. **Jinyuan Chen:** Managed the chicken animal model workflow. Analyzed tissue-specific target gene expression and co-wrote the Results and Discussion sections. **Jinjing Xu:** Analyzed raw sequencing data quality and optimized parameters for full-length transcript reconstruction. **Yingqiu Cai:** Analyzed genomic alignment of long-read transcripts and assessed structural mapping accuracy. **Yi Zong:** Analyzed differential expression patterns and interpreted transcript abundance variations under miR-22-3p knockdown. **Mengmeng Run:** Analyzed functional pathways and interpreted enrichment patterns related to muscle development. **Baoshun Qian:** Analyzed miR-22-3p target predictions and interpreted candidate gene regulatory roles in meat quality. **Qianhua Xie:** Analyzed alternative splicing patterns and interpreted splicing variations between the knockdown and control groups. **Xiaolu Luo:** Analyzed isoform switching events and interpreted functional consequences of altered isoform usage. **Beibei Liu:** Analyzed protein domain architecture of novel isoforms and interpreted structural implications. **Xingqing Zhang:** Analyzed protein-protein interaction networks and interpreted hub gene regulatory systems in muscle tissue. **Jiancheng Guo:** Designed and performed RT-PCR validation experiments and interpreted splicing confirmation results. **Fei Ye:** Analyzed bioinformatic workflow outputs and interpreted the biological coherence of computational findings. **Hai Xiang:** Interpreted physiological implications of transcriptomic findings in skeletal muscle development. **Hua Li:** Critically revised the scientific framework and optimized biological interpretations of genomic results. **Zheng Ma:** Conceived and designed the study, secured funding, supervised the research team, and finalized the manuscript.

## Ethical statement

All animal experiments were reviewed and approved by the Experimental Animal Welfare and Animal Experiment Ethics Committee of Foshan University (approval no. FOSU202103-28). All procedures involving animals were performed in strict accordance with the guidelines of the Chinese Animal Protection Association.

## Disclosures

The authors declare that they have no known competing financial interests or personal relationships that could have appeared to influence the work reported in this paper.

## Data Availability

The raw Oxford Nanopore long-read transcriptome sequencing data reported in this paper have been deposited in the Genome Sequence Archive (GSA) under accession number CRA049478 and are publicly accessible at .https://ngdc.cncb.ac.cn/gsa

## Footnotes

Supplementary material associated with this article can be found, in the online version, at doi:10.1016/j.psj.2026.107769.
