# Supplementary material for: Nanopore long-read transcriptome sequencing reveals miR-22-3p-mediated transcriptomic remodeling and alternative splicing in chicken pectoral muscle
Source: Poult Sci. 2026 Sep 13;105(12):107769. doi: 10.1016/j.psj.2026.107769 (PMC13625780; doi:10.1016/j.psj.2026.107769)
Supplement: Supplementary file 1 [file mmc1.docx]

**Description of Additional Supplementary Files**

**File name:** Supplementary Data 1

**Description**: List of differentially expressed transcripts (DETs) after miR-22-3p knockdown.

**File name:** Supplementary Data 2

**Description:** GO and KEGG enrichment analysis of differentially expressed transcripts.

**File name:** Supplementary Data 3

**Description:** Detailed alternative splicing events (ASEs) and Percent Spliced-In (PSI) values across all samples in chicken pectoral muscle.

**File name:** Supplementary Data 4

**Description:** Significant transcript isoform switching events and changes in isoform fraction (IF) values in chicken pectoral muscle responding to miR-22-3p knockdown.

**File name:** Supplementary Data 5

**Description**: List of differentially expressed genes (DEGs) after miR-22-3p knockdown.

**File name:** Supplementary Data 6

**Description:** List of miRDB predicted targets and PPI hub genes.
