# Supplementary material for: Nanopore long-read transcriptome sequencing reveals miR-22-3p-mediated transcriptomic remodeling and alternative splicing in chicken pectoral muscle
Source: Poult Sci. 2026 Sep 13;105(12):107769. doi: 10.1016/j.psj.2026.107769 (PMC13625780; doi:10.1016/j.psj.2026.107769)
Supplement: Supplementary file 2 [file mmc8.docx]

# Supplementary Information (SI)

**Nanopore long-read transcriptome sequencing reveals miR-22-3p-mediated transcriptomic remodeling and alternative splicing in chicken pectoral muscle**

**This file includes:**

**A.Supplementary tables**

Supplementary Tables A1–A2

**B.Supplementary figures**

Supplementary Figures B1–B3

*Note：*Supplementary Data 1–6 are provided as separate files.

A. **Supplementary tables**

**Table A1: Primers used for the validation of AS events*.***

| Gene ID | Forward primer | Reverse primer |
| --- | --- | --- |
| IDH3A | ACAGCAATCCAAGGACCAGG | GTTTTCTGCTGCTTCCCTGC |
| IDH3B | TGCCGAACCTCTATGGGAAC | TGAAATCCAGGGACGTGGC |
| LOC771456 | CCCCTGAGATGAAGAGAGCTGTA | TCTTTGTAGGCGGCCACG |
| LOC107049847 | GCCGCTGTTTCTCAGCTTTC | TGTCTCAGCTCACTGCATCC |
| EIF4EBP1 | GGAAAGCAGTTTCTATTGTTTTGGG | GAGAAGCACTCTCAGGGCAC |

**Table A2: Statistics of full-length sequence data*.***

| Sample ID | Number of clean reads  (excluding rRNA) | Number of full-length reads | Full-Length Percentage (FL%) |
| --- | --- | --- | --- |
| I1 | 5,258,027 | 4,634,899 | 88.15% |
| I2 | 5,000,080 | 4,428,998 | 88.58% |
| I3 | 4,822,332 | 4,224,026 | 87.59% |
| NC1 | 4,652,940 | 4,120,664 | 88.56% |
| NC2 | 5,910,819 | 5,131,777 | 86.82% |
| NC3 | 5,779,117 | 5,113,961 | 88.49% |

***Note:*** Sample ID: sample name; Number of clean reads (excluding rRNA): number of clean reads after filtering ribosomal RNA; Number of full-length reads: number of full-length reads; Full-Length Percentage (FL%): proportion of full-length reads.

B. **Supplementary figures**

**Fig. B1. Classification and count distribution of identified transcripts and genes.**

(a) Quantity distribution of annotated transcripts (n=10,030) and novel transcripts (n=24,727) identified in the full-length transcriptome (total n=34,757). (b) Quantity distribution of annotated genes (n=9,482) and novel genes (n=8,687) retained for downstream functional genomic and differential expression analyses (total n=18,169)

**Fig. B2. Significantly Enriched Muscle Biology-Related Pathways of Known Transcripts of Known Genes.**

**Fig. B3. Venn diagram illustrating overlaps between miRDB-predicted miR-22-3p targets and PPI hub genes of chicken pectoral muscle DEGs.**
